# Supplementary material for: Endosomal trafficking defects alter neural progenitor proliferation and cause microcephaly
Source: Nat Commun. 2022 Jan 10;13:16. doi: 10.1038/s41467-021-27705-7 (PMC8748540; doi:10.1038/s41467-021-27705-7)
Supplement: Supplementary file 1 — Supplementary Information [file 41467_2021_27705_MOESM1_ESM.pdf]

Endosomal trafficking defects alter neural progenitor proliferation and cause microcephaly

Jacopo A. Carpentieri[1], Amandine Di Cicco[1], Marusa Lampic[1], David Andreau[1], Laurence Del Maestro[2], Fatima El Marjou[1], Laure Coquand[1], Nadia Bahi-Buisson[3,4], Jean-Baptiste Brault[1], Alexandre D. Baffet[1,5,#]

Supplementary Information

Supplementary Figure 1

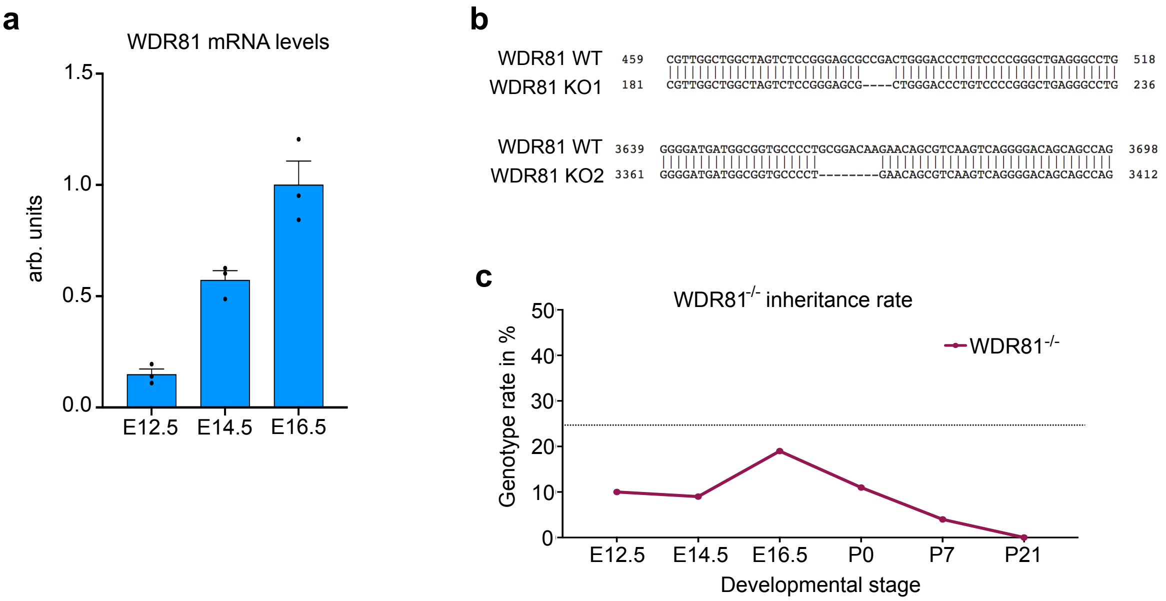

Supplementary Fig.1. Expression of WDR81 and description of WDR81$^{-/-}$ mice.

a. Expression of WDR81 (isoform 1) in E12.5, E14.5 and E16.5 cortices, measured by QPCR. **b.** Sequencing of WDR81 knock-out animals reveals a 4 base pair deletion in KO1 and an 8 base pair deletion in KO2, both leading to frameshifts and premature STOP codons. **c.** Rate of WDR81$^{-/-}$ embryos and pups recovered throughout time. The expected rate is 25% (dashed line). By P21, no mutant was detected. Data are expressed as mean +/- standard deviation (SD).

Supplementary Figure 2

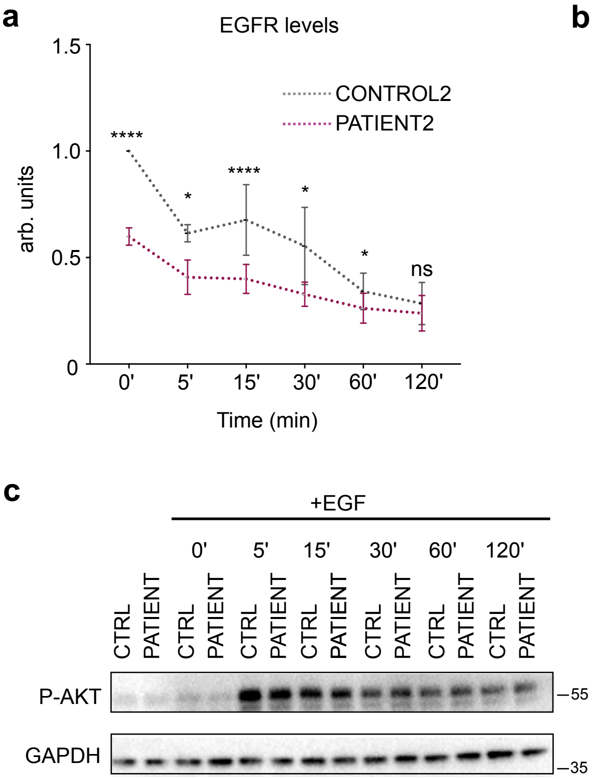

Supplementary figure 2. EGFR, P-ERK and P-AKT levels in Control and WDR81 patient cell lines.

a. Quantification of EGFR levels, normalized to control levels at T0 for Control-2 and WDR81 patient-2 cell lines (T0 p<0,0001; T5 p=0,0164; T15 p<0,0001; T30 p=0,0301; T60 p=0,303) (n=5 independent experiments). **b.** Quantification of P-ERK levels, normalized to control levels at T5 for Control-2 and WDR81 patient-2 cell lines (T15 p=0,0013; T30 p=0,0376) (n=5 independent experiments). *p<0,05; **p<0,01; ***p<0,001; ****p<0,0001 by two-tailed unpaired t-tests. **c.** Time course of P-AKT levels in control-1 and WDR81 patient-1 fibroblasts following an EGF pulse (n=3 independent experiments). All data are expressed as mean +/- standard deviation (SD).

Supplementary Figure 3

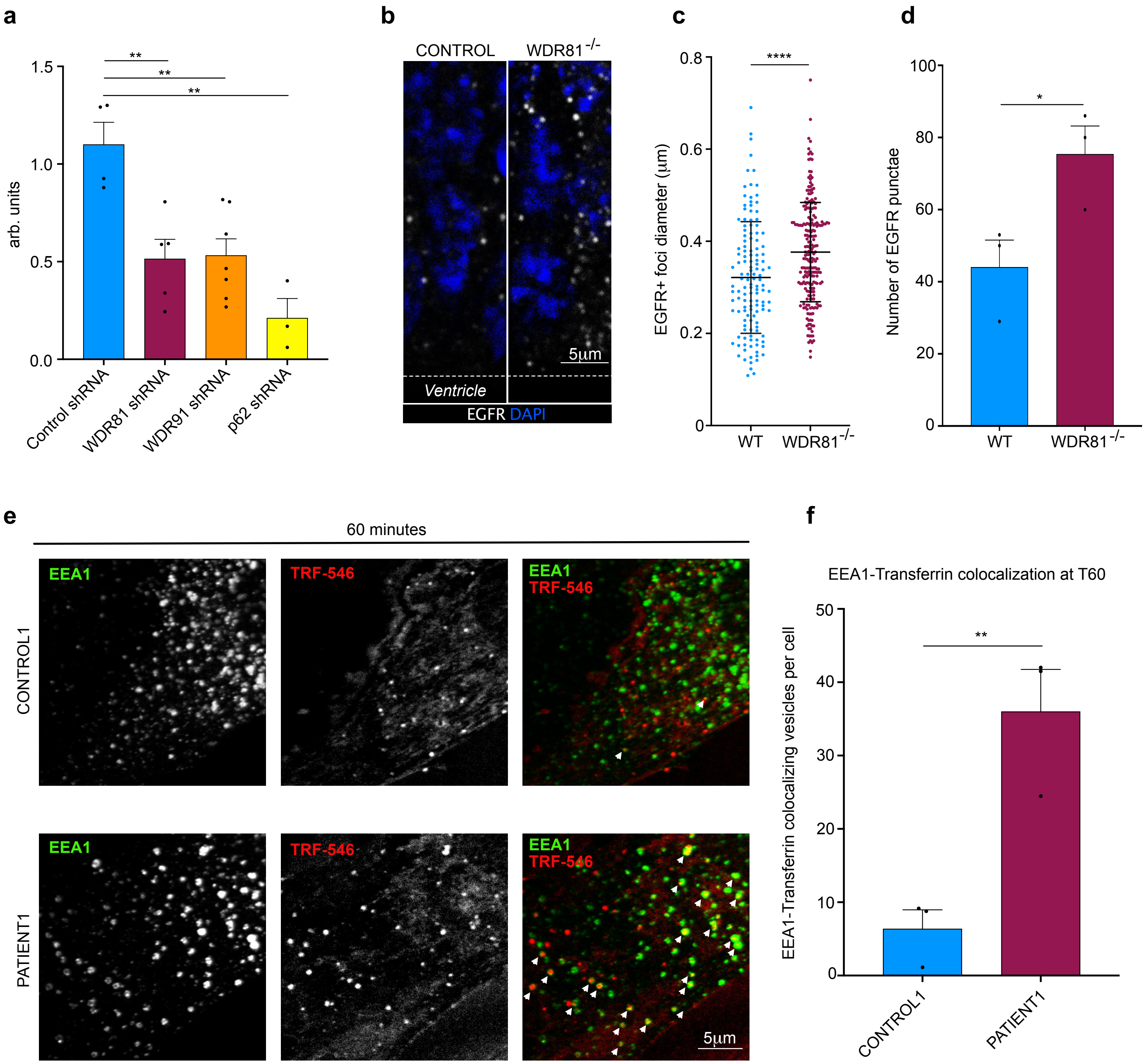

Supplementary figure 3. KD validation, EGFR intracellular accumulation *in vivo* and recycling assay

a. QPCR validation of WDR81 (n=3, p=0,062), WDR91 (n=7, p=0,0031) and p62 (n=3, p=0,0025) shRNA-mediated knockdown in Neuro2A cells. **b.** Ventricular zone of E14.5 WT and WDR81$^{-/-}$ mice cortices stained for EGFR. **c.** Quantification of individual EGFR+ foci in WT and WDR81$^{-/-}$ VZ in 200x100 μm crops reveals increased size in mutant brains (n=3 independent brains per genotype, p<0,0001). **d.** Quantification in WT and WDR81$^{-/-}$ VZ in 200x100 μm crops reveals increased number of EGFR foci in mutant brains (n=3 independent brains per genotype, p=0,0452). **e.** Transferrin^{546} uptake assay in control and WDR81 patient fibroblasts stained for EEA1. White arrowheads indicate colocalizing foci **f.** Quantification of transferrin^{546} and EEA1 colocalization at 60 minutes after transferrin^{546} pulse reveals prolonged localization within early endosomes of WDR81 patient cells (n=3 independent experiments, p=0,0094). All data are expressed as mean +/- standard deviation (SD). *p<0,05; **p<0,01; ****p<0,0001 by two-tailed unpaired t-test (A, D & F) and Mann-Whitney tests (C).
