# Supplementary material for: Endosomal trafficking defects alter neural progenitor proliferation and cause microcephaly
Source: Nat Commun. 2022 Jan 10;13:16. doi: 10.1038/s41467-021-27705-7 (PMC8748540; doi:10.1038/s41467-021-27705-7)
Supplement: Supplementary file 3 — Reporting Summary [file 41467_2021_27705_MOESM3_ESM.pdf]

nature research

Corresponding author(s): Alexandre BAFFET

Last updated by author(s): 2021/06/01

# Reporting Summary

Nature Research wishes to improve the reproducibility of the work that we publish. This form provides structure for consistency and transparency in reporting. For further information on Nature Research policies, see our Editorial Policies and the Editorial Policy Checklist.

## Statistics

For all statistical analyses, confirm that the following items are present in the figure legend, table legend, main text, or Methods section.

| n/a | Confirmed | |
|---|---|---|
| ☐ | ☒ | The exact sample size (*n*) for each experimental group/condition, given as a discrete number and unit of measurement |
| ☐ | ☒ | A statement on whether measurements were taken from distinct samples or whether the same sample was measured repeatedly |
| ☐ | ☒ | The statistical test(s) used AND whether they are one- or two-sided<br>*Only common tests should be described solely by name; describe more complex techniques in the Methods section.* |
| ☒ | ☐ | A description of all covariates tested |
| ☒ | ☐ | A description of any assumptions or corrections, such as tests of normality and adjustment for multiple comparisons |
| ☐ | ☒ | A full description of the statistical parameters including central tendency (e.g. means) or other basic estimates (e.g. regression coefficient) AND variation (e.g. standard deviation) or associated estimates of uncertainty (e.g. confidence intervals) |
| ☐ | ☒ | For null hypothesis testing, the test statistic (e.g. *F*, *t*, *r*) with confidence intervals, effect sizes, degrees of freedom and *P* value noted<br>*Give P values as exact values whenever suitable.* |
| ☒ | ☐ | For Bayesian analysis, information on the choice of priors and Markov chain Monte Carlo settings |
| ☒ | ☐ | For hierarchical and complex designs, identification of the appropriate level for tests and full reporting of outcomes |
| ☒ | ☐ | Estimates of effect sizes (e.g. Cohen's *d*, Pearson's *r*), indicating how they were calculated |

*Our web collection on statistics for biologists contains articles on many of the points above.*

## Software and code

Policy information about availability of computer code

| | |
|---|---|
| Data collection | Microscopes were all equiped with Metamorph software. |
| Data analysis | All images were analyzed using Fiji software. Data were analyzed using Prsim 9. |

For manuscripts utilizing custom algorithms or software that are central to the research but not yet described in published literature, software must be made available to editors and reviewers. We strongly encourage code deposition in a community repository (e.g. GitHub). See the Nature Research guidelines for submitting code & software for further information.

## Data

Policy information about availability of data

All manuscripts must include a data availability statement. This statement should provide the following information, where applicable:

- Accession codes, unique identifiers, or web links for publicly available datasets
- A list of figures that have associated raw data
- A description of any restrictions on data availability

The original pictures of WB membranes are provided in the file "Supplementary information_WB membranes".The immunofluorescence data that support the findings of this study, due to their large size, are available from the corresponding author upon reasonable request.

# Field-specific reporting

Please select the one below that is the best fit for your research. If you are not sure, read the appropriate sections before making your selection.

☒ Life sciences ☐ Behavioural & social sciences ☐ Ecological, evolutionary & environmental sciences

For a reference copy of the document with all sections, see nature.com/documents/nr-reporting-summary-flat.pdf

# Life sciences study design

All studies must disclose on these points even when the disclosure is negative.

| | |
|---|---|
| Sample size | No sample size calculation was performed. We followed standard approaches and sample size was usually 3-5, which is very classic in the field of neurodevelopment. Higher sample size is challenging for in vivo studies and significant results are clear after three independent repetitions, especially for strong phenotypes such as observed here. |
| Data exclusions | No data was excluded from the analysis |
| Replication | All experiments were replicated at least three independent times. All "n's" is this study represent independent repetitions. All replications were successful. |
| Randomization | There was no randomization, WT and mutant samples were compared. |
| Blinding | Analysis were not performed blindly, as mutant samples were easily identifiable (smaller brain, swollen endosomes...). Key experiments were analyzed independently by different researchers, leading to the same conclusion. |

# Reporting for specific materials, systems and methods

We require information from authors about some types of materials, experimental systems and methods used in many studies. Here, indicate whether each material, system or method listed is relevant to your study. If you are not sure if a list item applies to your research, read the appropriate section before selecting a response.

## Materials & experimental systems

| n/a | Involved in the study | |
|---|---|---|
| ☐ | ☒ | Antibodies |
| ☐ | ☒ | Eukaryotic cell lines |
| ☒ | ☐ | Palaeontology and archaeology |
| ☐ | ☒ | Animals and other organisms |
| ☒ | ☐ | Human research participants |
| ☒ | ☐ | Clinical data |
| ☒ | ☐ | Dual use research of concern |

## Methods

| n/a | Involved in the study | |
|---|---|---|
| ☒ | ☐ | ChIP-seq |
| ☒ | ☐ | Flow cytometry |
| ☒ | ☐ | MRI-based neuroimaging |

## Antibodies

| | |
|---|---|
| Antibodies used | Primary antibodies used: mouse anti Ctip-2 (Abcam ab18465), rabbit anti Pax6 (Biolegend 901301), Sheep anti TBR2/EOMES (R&D system AF6166), rabbit anti NEUN (Abcam ab177487), goat anti Phospho Histone3 (Santa Cruz SC-12927), rabbit anti BRDU (Abcam AB152095), rabbit cleaved caspase-3(CST 3661), rabbit anti CUX-1 (Santa Cruz, discontinued), rabbit anti Ki67 (abcam ab15580), rabbit anti EGFR (CST 4267), mouse anti p-ERK (CST 9106), rabbit anti GAPDH (Sigma AldrichG9545), anti p-AKT (CST 4060) and mouse anti EEA-1 (BD biosciences 610457). Secondary antibodies used: donkey Alexa Fluor 488 anti-mouse, anti-rabbit, anti-goat (Jackson laboratories 715-545-150, 711-165-152, 715-605-152), donkey Alexa Fluor 555 anti-mouse, anti-rabbit, anti-goat (Jackson laboratories 715-545-150, 711-165-152, 715-605-152), donkey Alexa Fluor 647 anti-mouse, anti-rabbit, anti-goat (Jackson laboratories 715-545-150, 711-165-152, 715-605-152). |
| Validation | All antibodies were validated based on expected subcellular localization or molecular weight of the detected protein. All antibodies are routinely used in the laboratory or, for phsopho-specific antibodies, in the cell cycle field.<br>For mouse anti Ctip-2 (Abcam ab18465), antibody was validated by IF and WB (https://www.abcam.com/ctip2-antibody-25b6-ab18465.html). For rabbit anti Pax6 (Biolegend 901301), antibody was validated by IF and IHC (https://www.biolegend.com/en-us/products/purified-anti-pax-6-antibody-11511?GroupID=GROUP26). For Sheep anti TBR2/EOMES (R&D system AF6166), antibody was validated by WB and IF (https://www.rndsystems.com/products/human-eomes-antibody_af6166). For rabbit anti NEUN (Abcam ab177487) antibody was validated by WB and IF (https://www.abcam.com/neun-antibody-epr12763-neuronal-marker-ab177487.html#lb). For goat anti Phospho Histone3 (Santa Cruz SC-12927) antibody was validated using WB and IF (https://antibodyregistry.org/search.php?q=AB_2233069). For rabbit anti BRDU (Abcam AB152095) antibody was validated using IF (https://www.abcam.com/brdu-antibody-ab152095.html). For rabbit cleaved caspase-3(CST 9661), antibody was validated using WP and IHC (https://www.cellsignal.com/products/primary-antibodies/cleaved-caspase-3-asp175-antibody/9661). For rabbit anti Ki67 (abcam ab15580), antibody was validated using IF and IHC (https://www.abcam.com/ki67-antibody-ab15580.html#lb). For rabbit anti EGFR |

(CST 4267) antibody was validated using WB and IF (https://www.cellsignal.com/products/primary-antibodies/egf-receptor-d38b1-xp-rabbit-mab/4267). For mouse anti p-ERK (CST 9106) antibody was validated using WB (https://www.cellsignal.com/products/primary-antibodies/phospho-p44-42-mapk-erk1-2-thr202-tyr204-e10-mouse-mab/9106). For rabbit anti GAPDH (Sigma AldrichG9545) antibody was validated using WB (https://www.sigmaaldrich.com/FR/fr/product/sigma/g9545).For anti p-AKT (CST 4060) antibody was validated using IF, IHC and WB (https://www.cellsignal.com/products/primary-antibodies/phospho-akt-ser473-d9e-xp-rabbit-mab/4060). For mouse anti EEA-1 (BD biosciences 610457), antibody was validated using WB and IF (https://www.bdbiosciences.com/en-fr/products/reagents/microscopy-imaging-reagents/immunofluorescence-reagents/purified-mouse-anti-eea1.610457).

## Eukaryotic cell lines

Policy information about cell lines

| | |
|---|---|
| Cell line source(s) | Patient and control fibroblasts were obtained by Nadia Bahi-Buisson (author on the manuscript) and described in Cavallin et al, Brain, 2017. Neuro2A were from ATCC (https://www.atcc.org/products/ccl-131). |
| Authentication | Mutant cell lines were sequenced at the mutation region |
| Mycoplasma contamination | Mycoplasma tests were performed weekly |
| Commonly misidentified lines (See ICLAC register) | All cell lines tested negative for mycoplasma. |

## Animals and other organisms

Policy information about studies involving animals; ARRIVE guidelines recommended for reporting animal research

| | |
|---|---|
| Laboratory animals | Mouse, Mus musculus, C57BL/6J and B6D2F1. Pregnant females. 10-30 week-old. |
| Wild animals | This study did not use wild animals |
| Field-collected samples | This study did not collect samples in the field |
| Ethics oversight | All experiments involving mice were carried out according to the recommendations of the European Community (2010/63/UE). The animals were bred and cared for in the Specific Pathogen Free (SPF) Animal Facility of Institut Curie (agreement C 75-05-18). All animal procedures were approved by the ethics committee of the Institut Curie CEEA-IC #118 and by French Ministry of Research (2016-002. |

Note that full information on the approval of the study protocol must also be provided in the manuscript.
