# Supplementary material for: Endosomal trafficking defects alter neural progenitor proliferation and cause microcephaly
Source: Nat Commun. 2022 Jan 10;13:16. doi: 10.1038/s41467-021-27705-7 (PMC8748540; doi:10.1038/s41467-021-27705-7)

Figure 3E

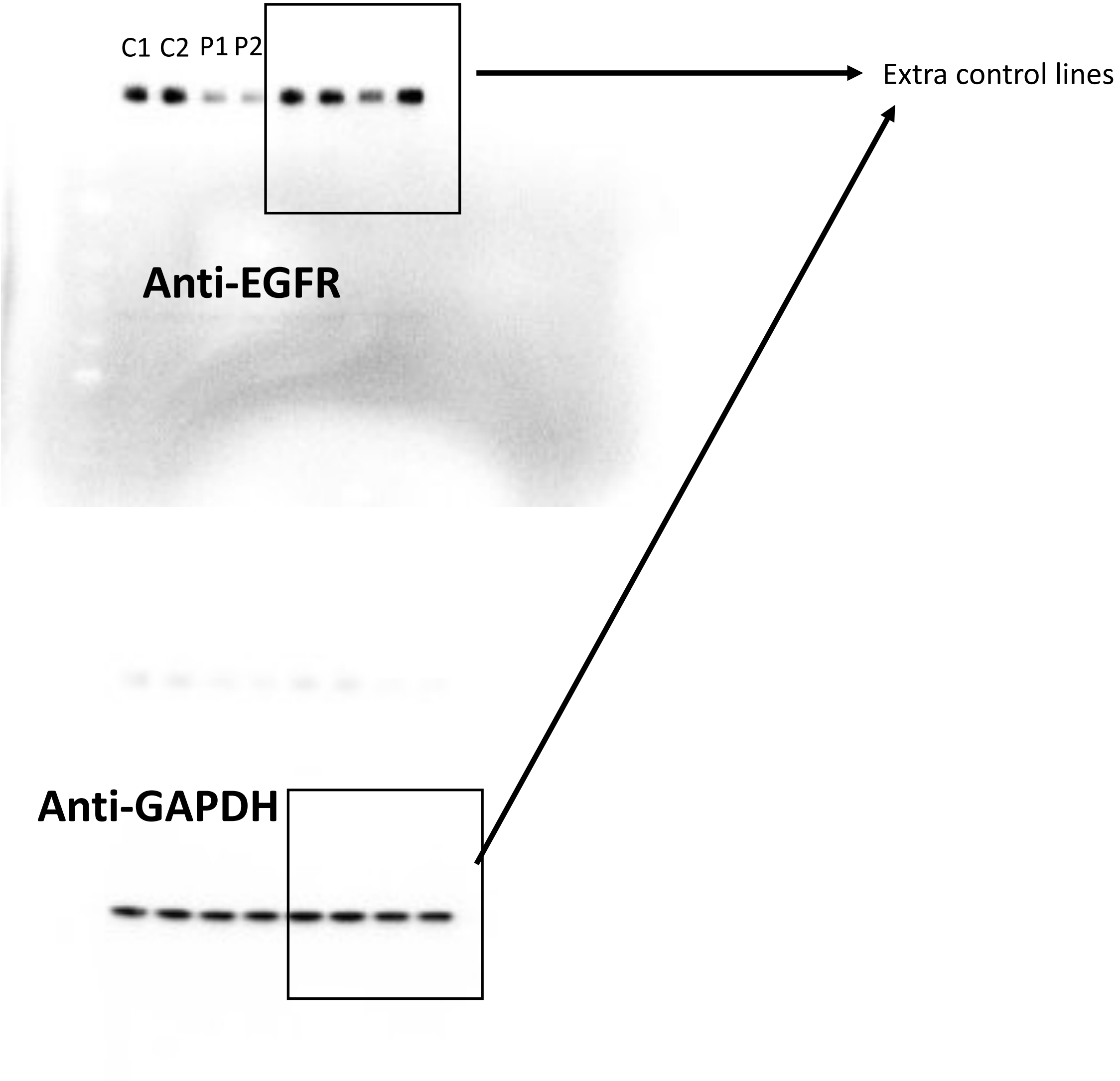

Figure 3G, H, I (replica 1)

C1 Vs P1

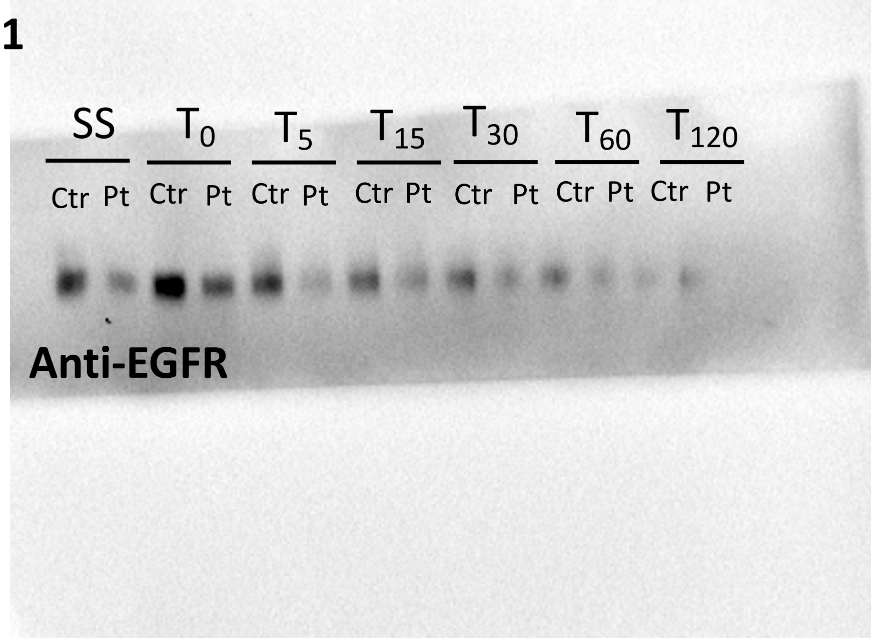

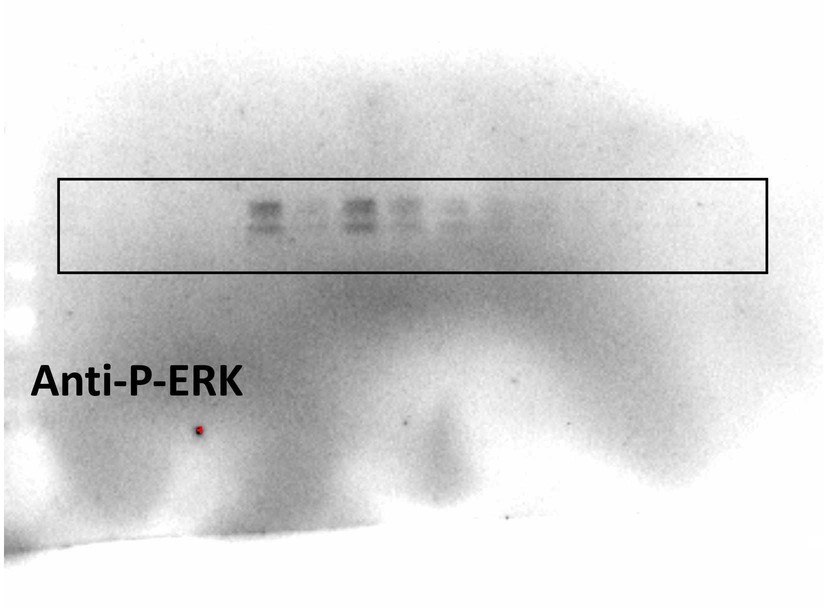

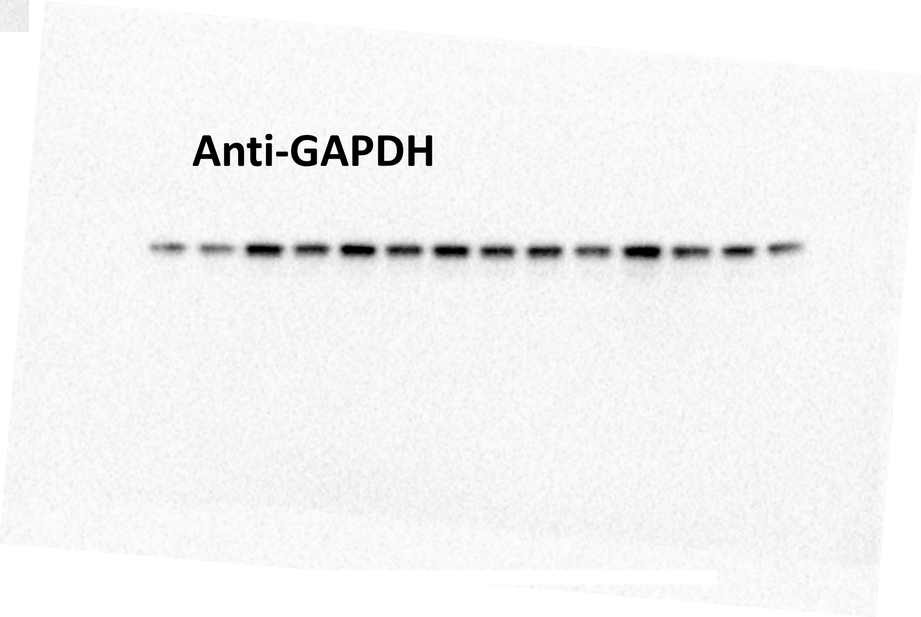

Figure 3G, H, I (replica 2)

C1 Vs P1

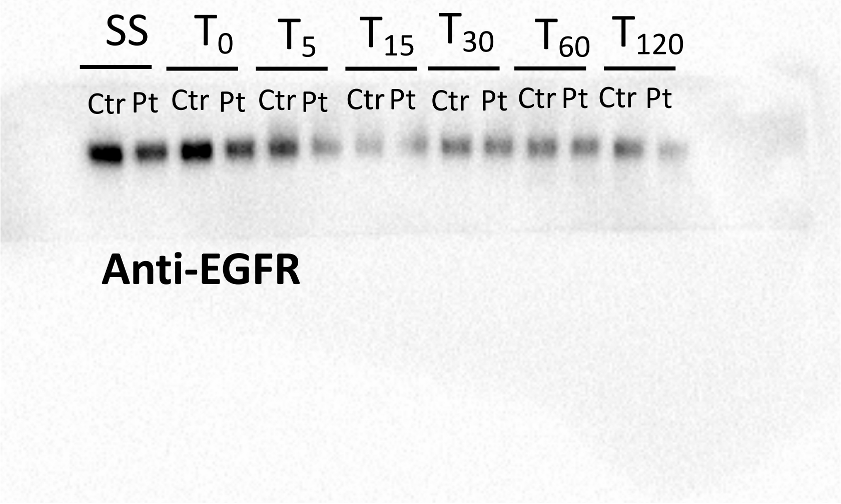

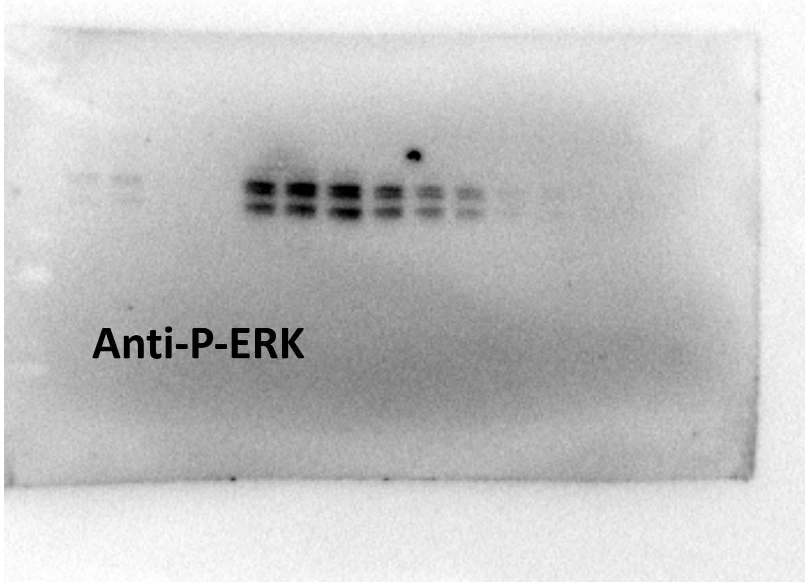

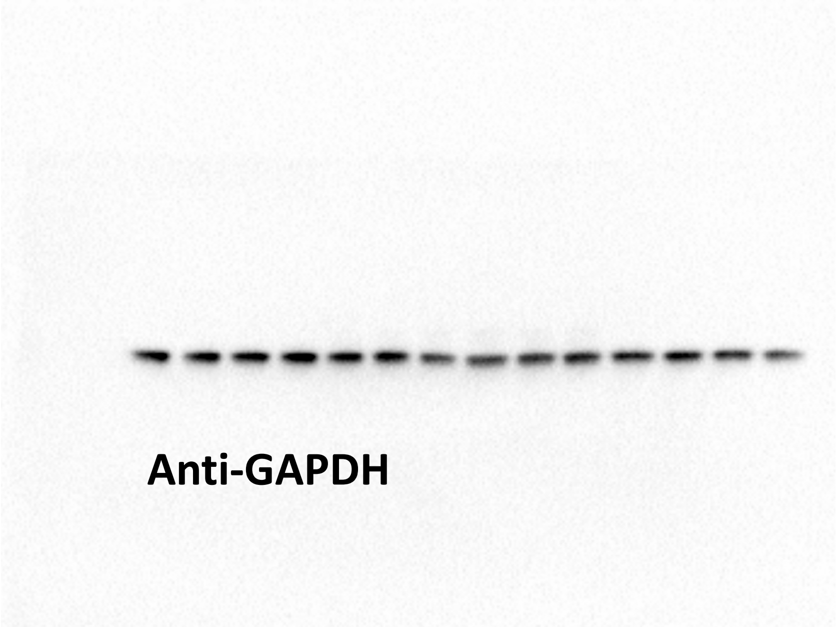

Figure 3G, H, I (replica 3)

C1 Vs P1

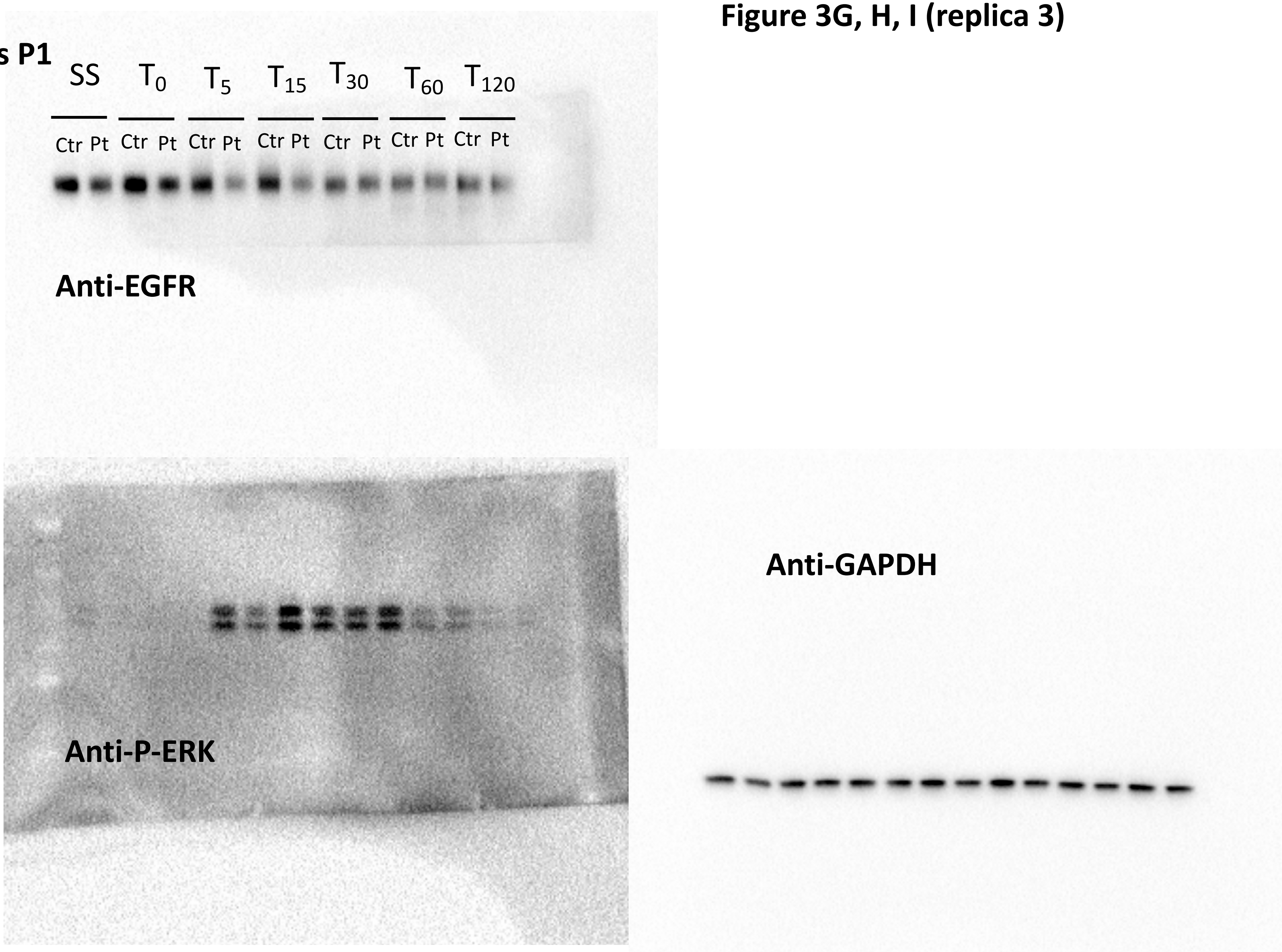

Figure 3G, H, I (replica 4)

C1 Vs P1

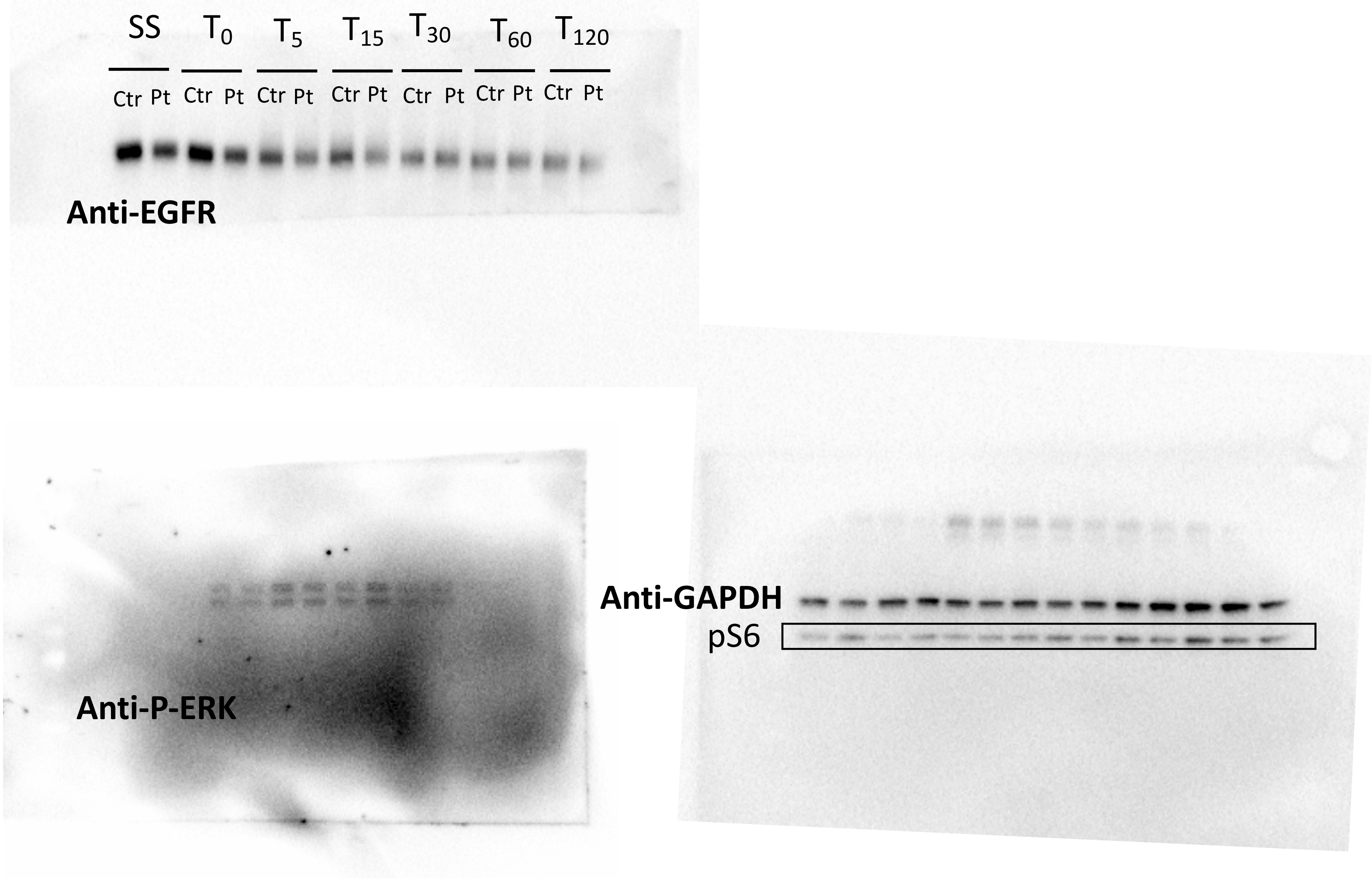

Figure 3G, H, I (replica 5)

C1 Vs P1

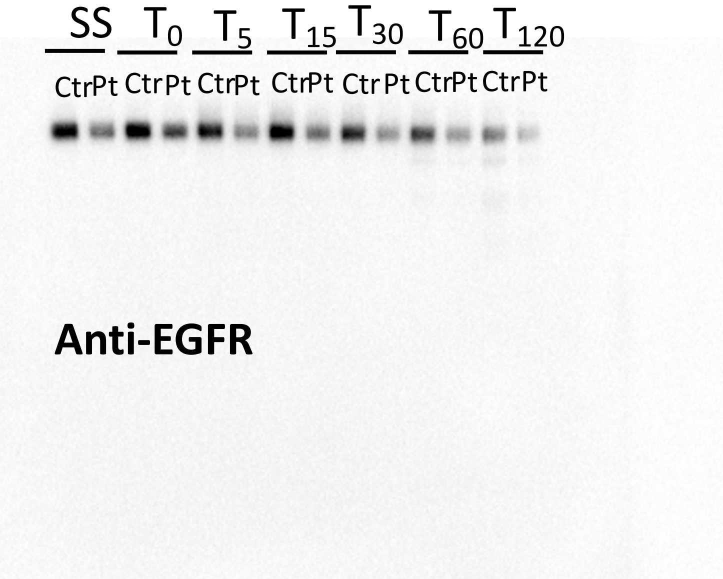

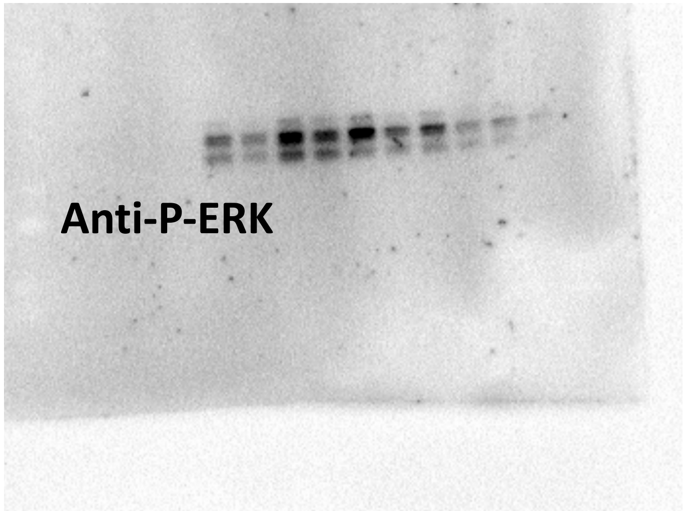

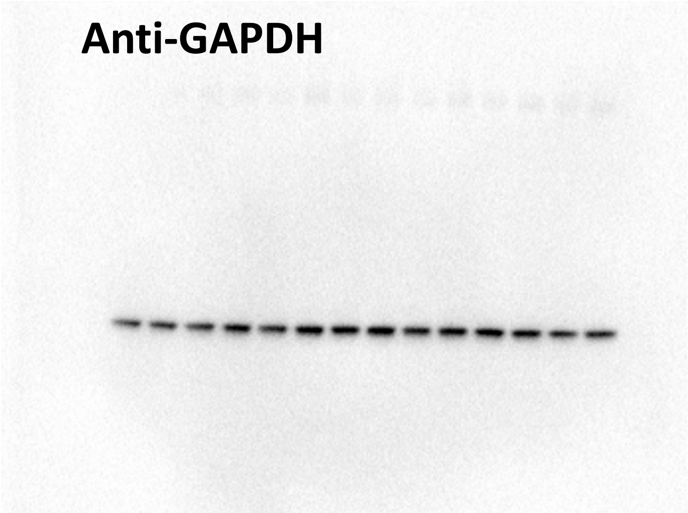

C2 Vs P2

Figure S2A, S2B (replica 1)

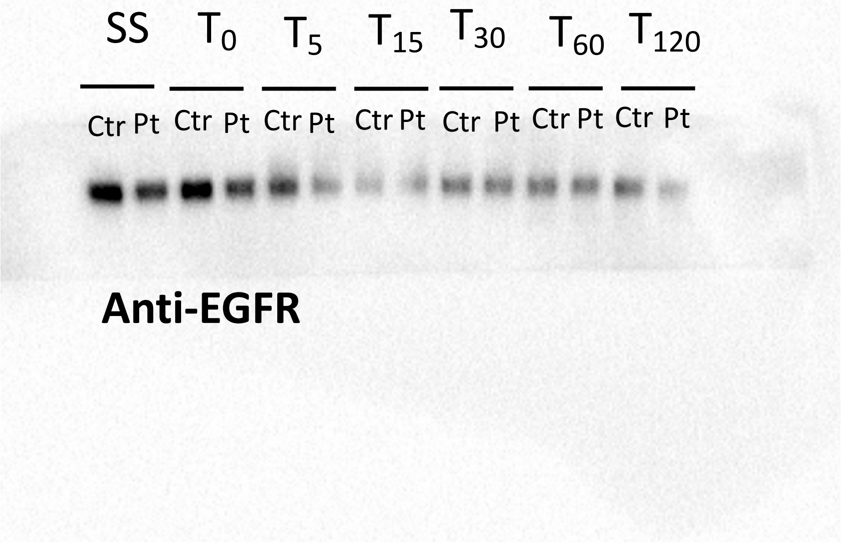

Anti-EGFR

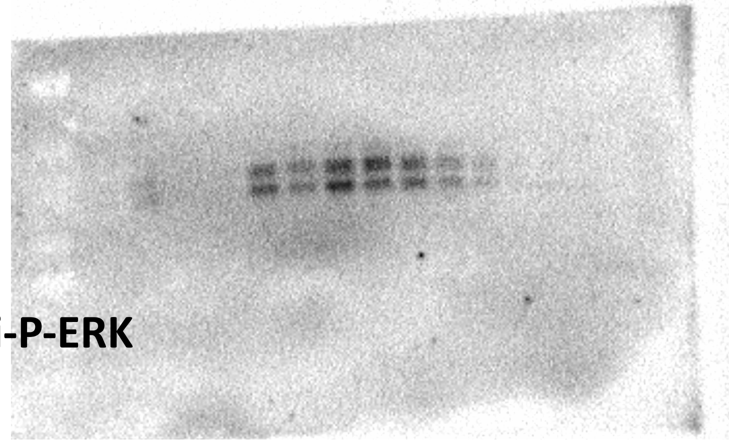

Anti-P-ERK

Anti-GAPDH

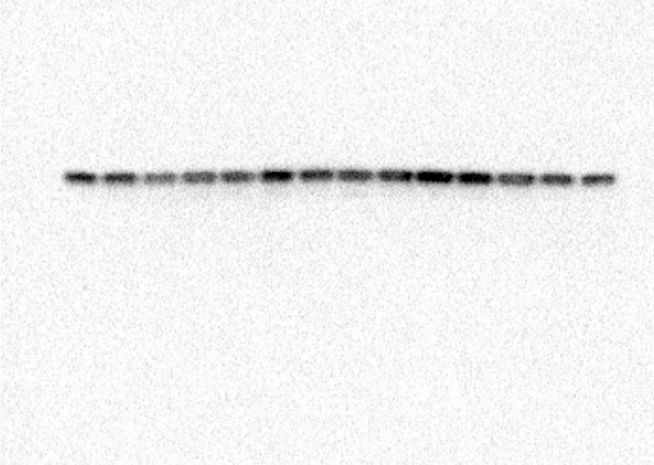

C2 Vs P2

Figure S2A, S2B (replica 2)

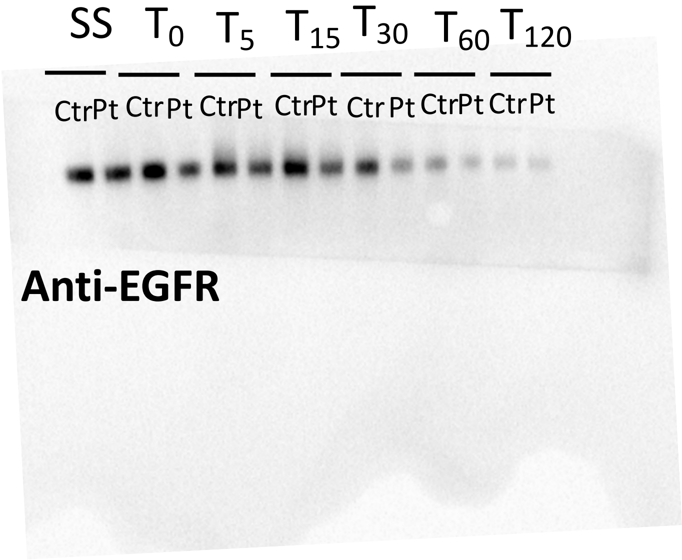

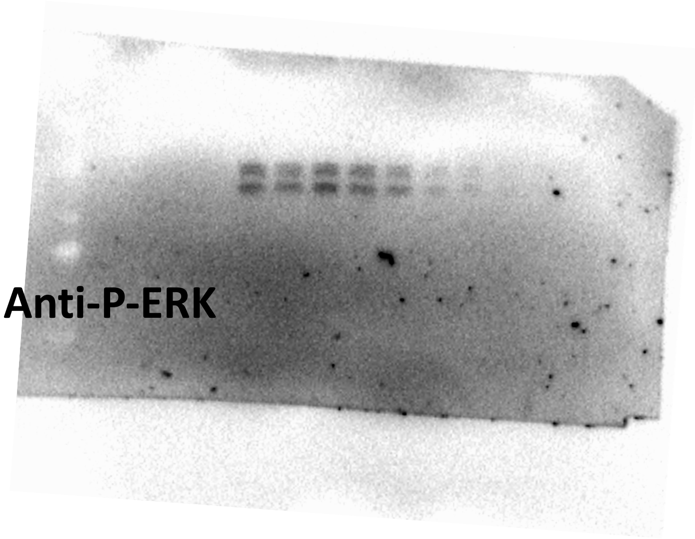

Anti-GAPDH

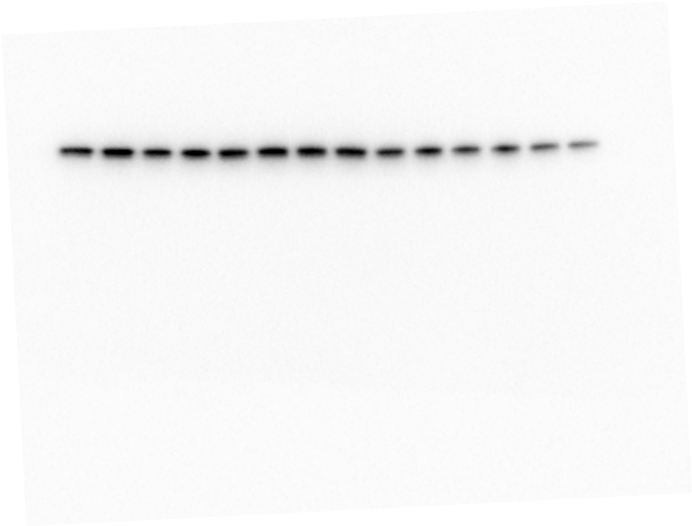

C2 Vs P2

Figure S2A, S2B (replica 3)

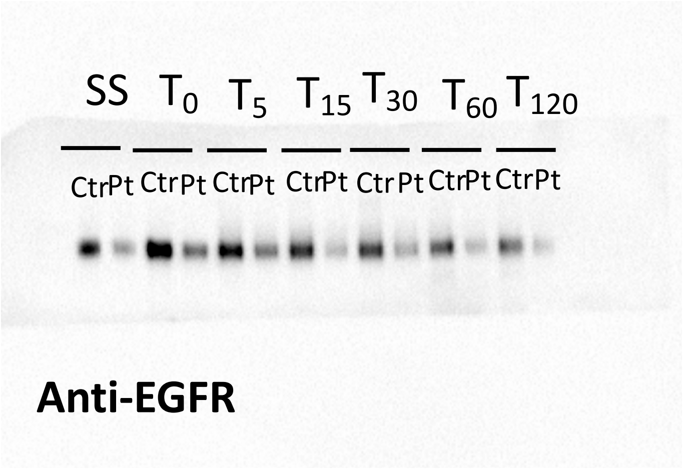

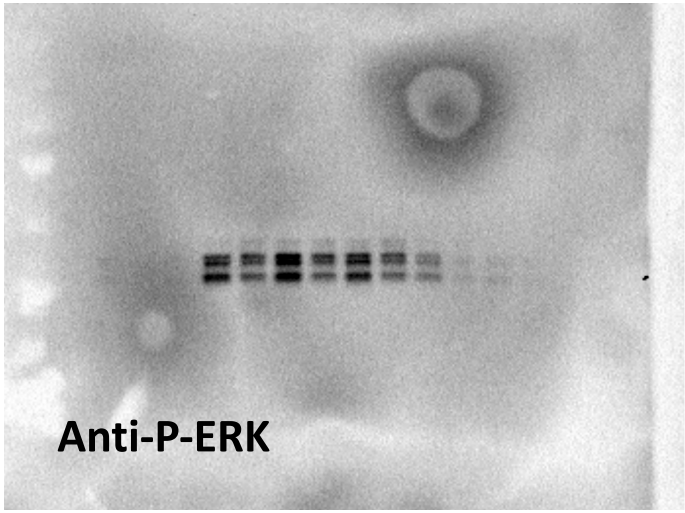

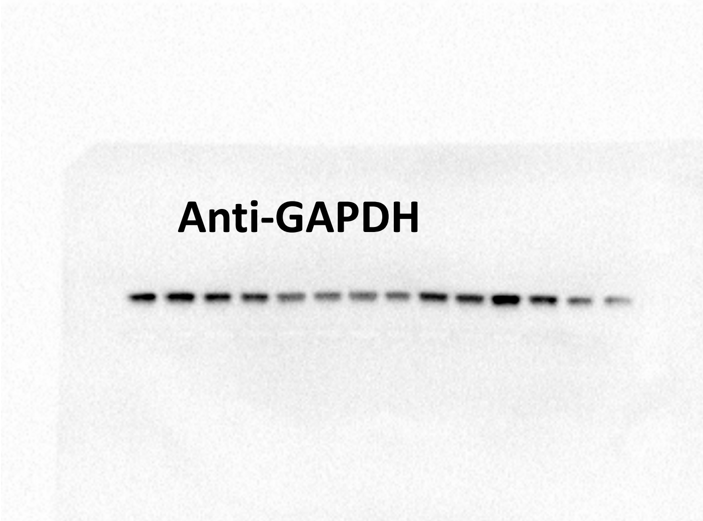

C2 Vs P2

Figure S2A, S2B (replica 4)

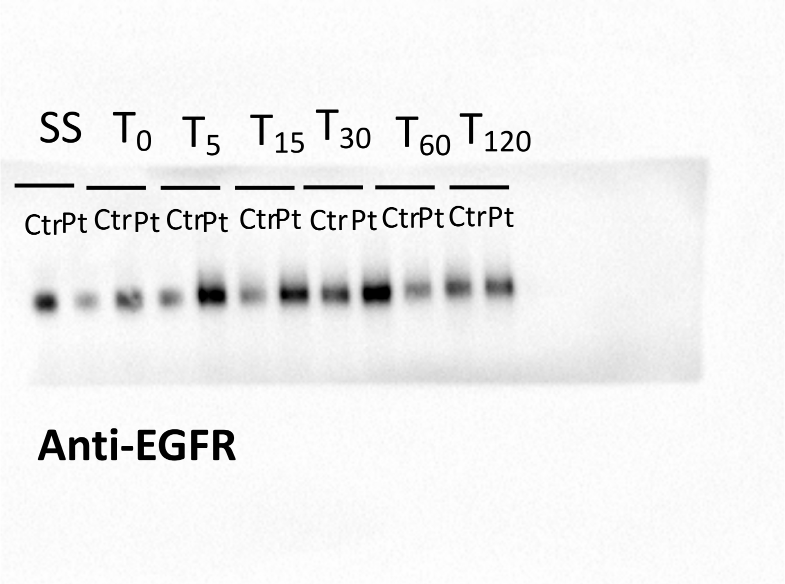

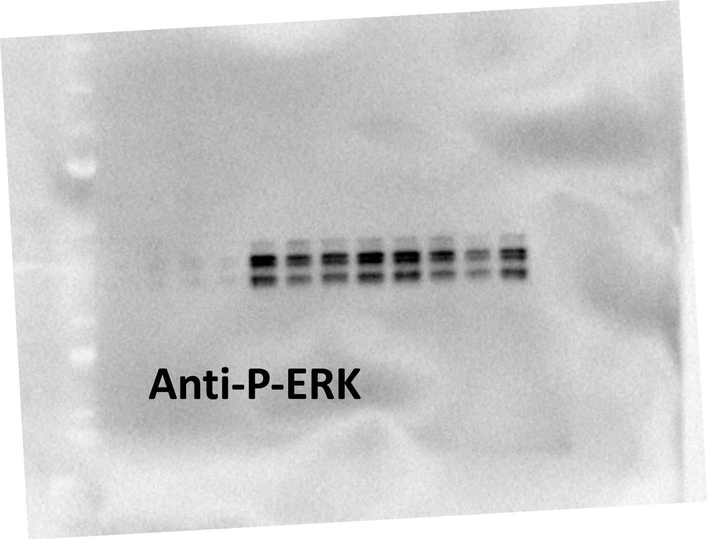

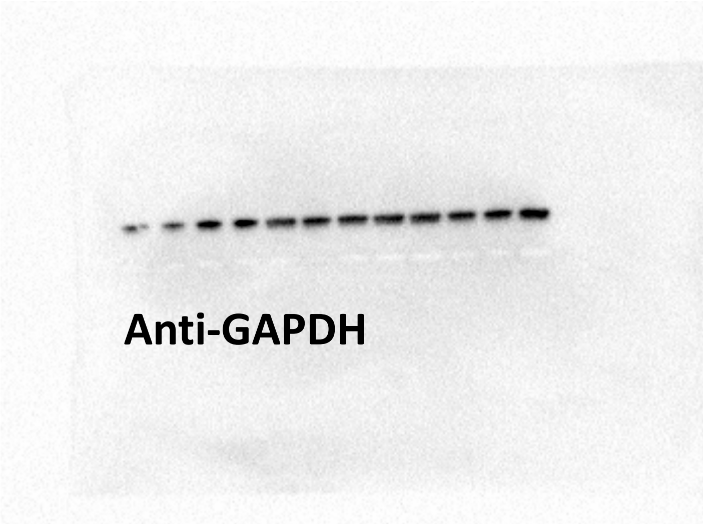

C2 Vs P2

Figure S2A, S2B (replica 5)

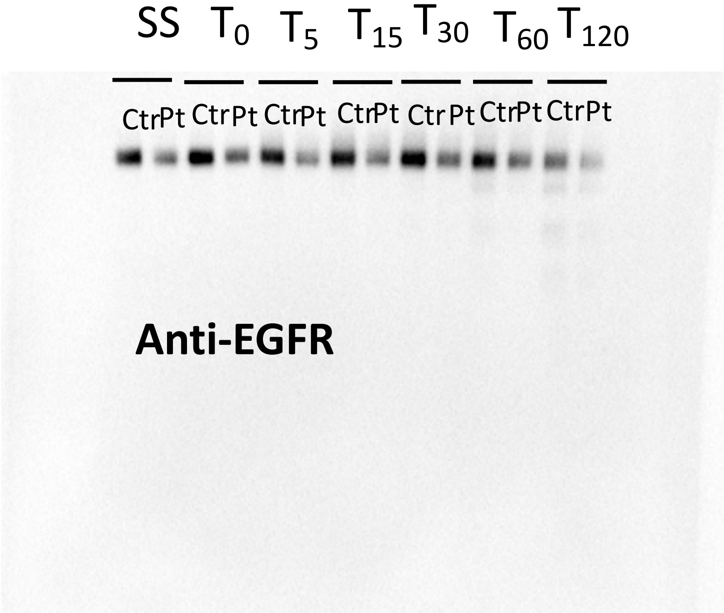

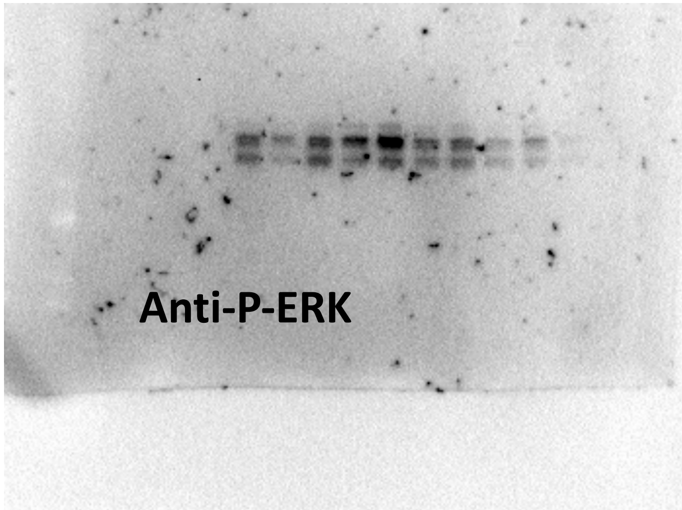

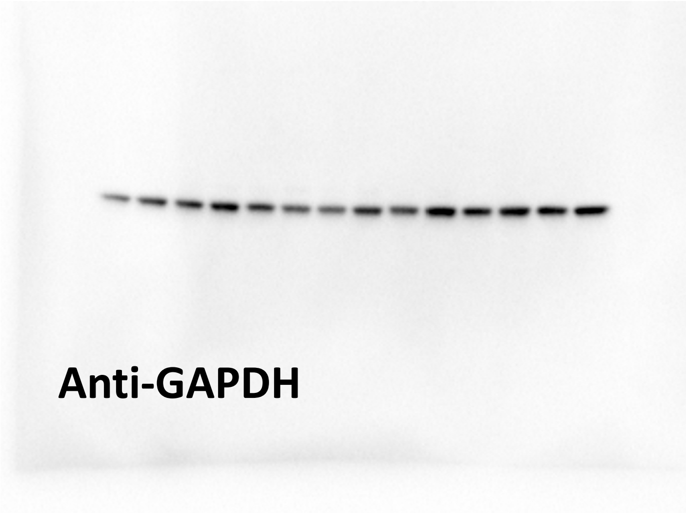

C1 Vs P1

Figure S3C (replica 1)

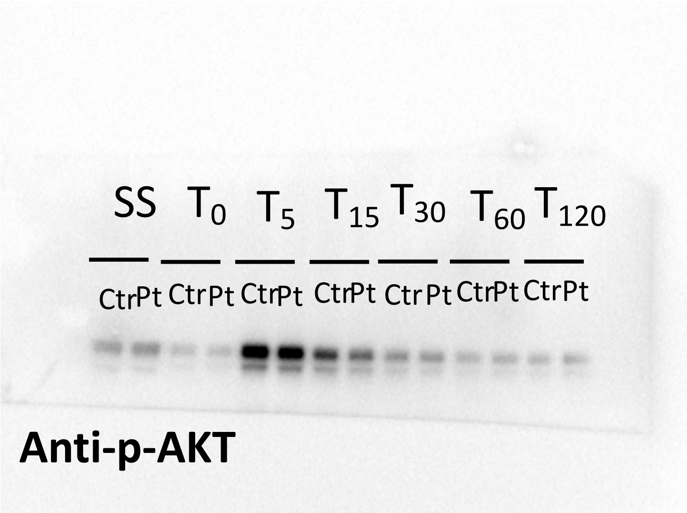

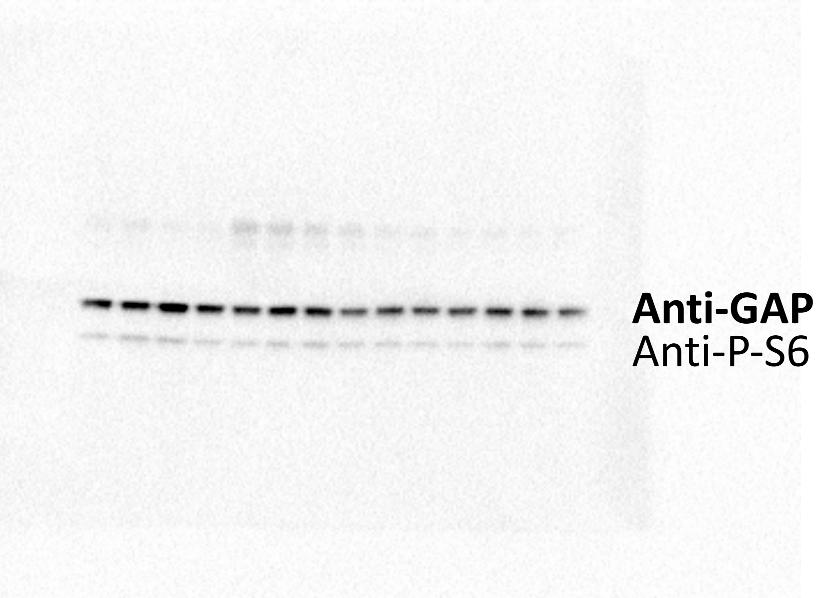

Anti-GAPDH
Anti-P-S6

C1 Vs P1

Figure S3C (replica 2)

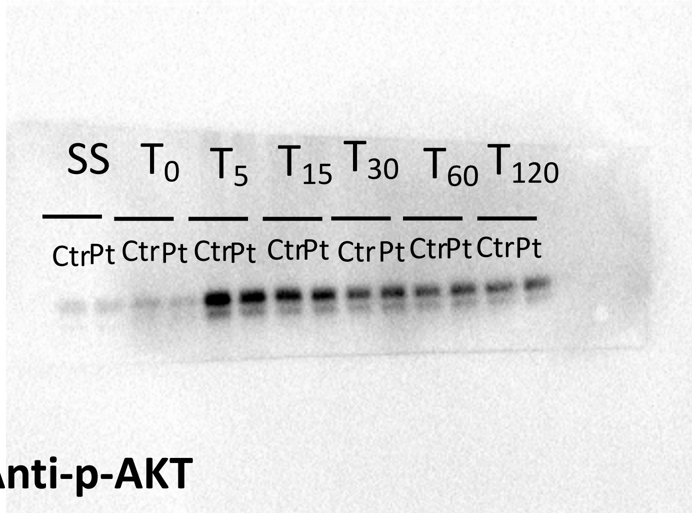

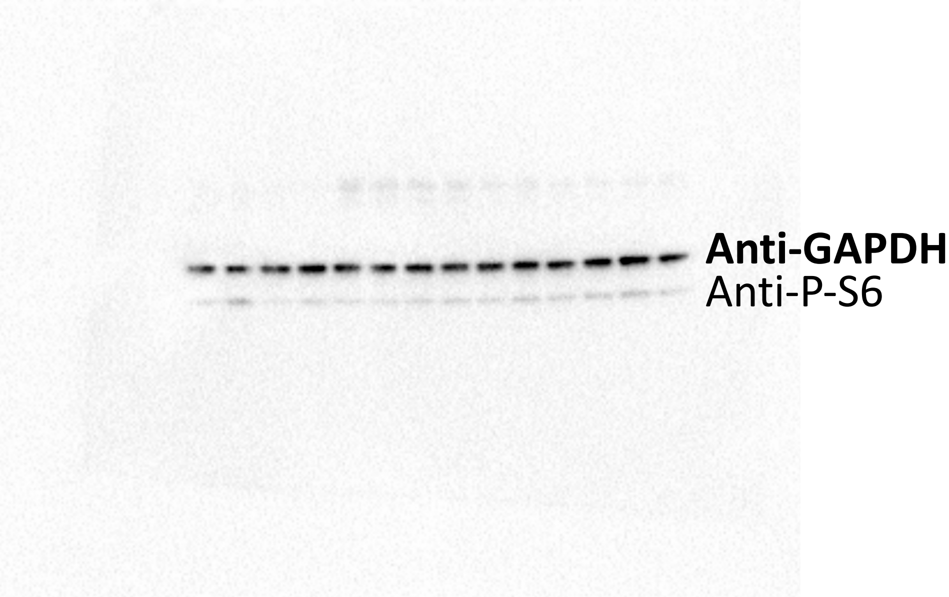

C1 Vs P1

Figure S3C (replica 3)

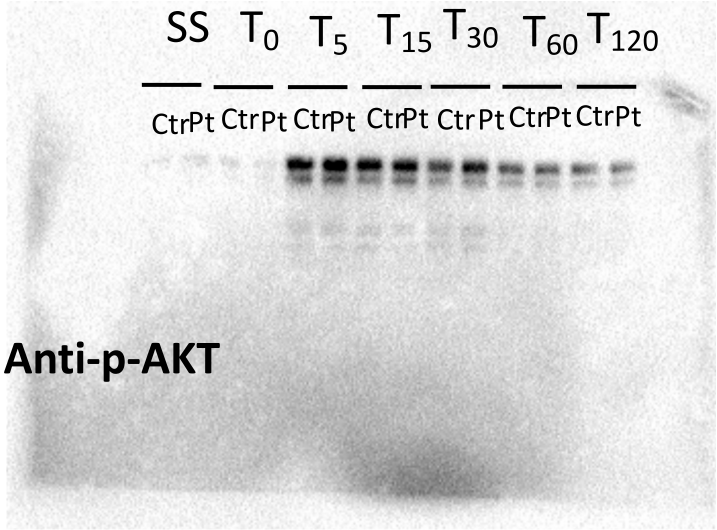

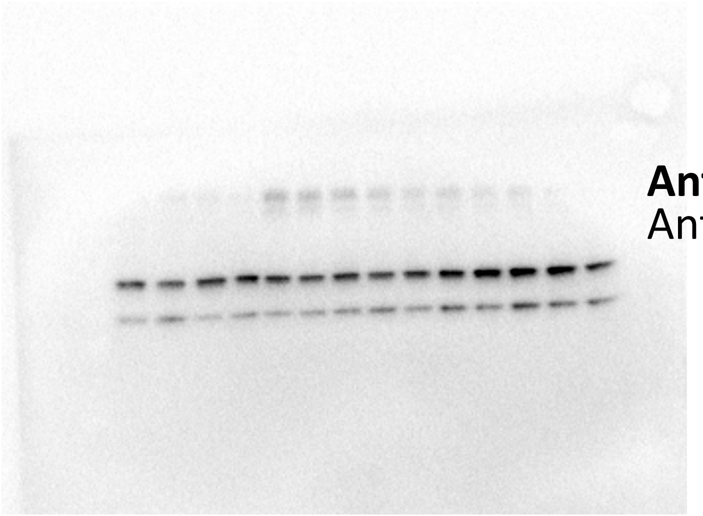

Anti-GAPDH
Anti-P-S6

C2 Vs P2

Figure S3C (replica 1)

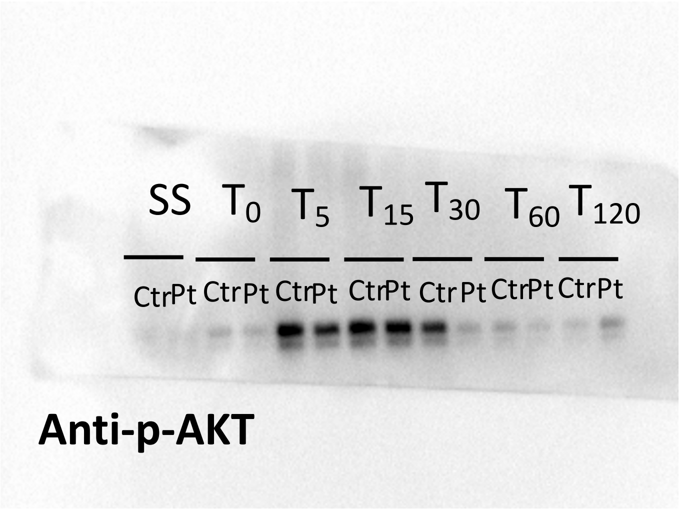

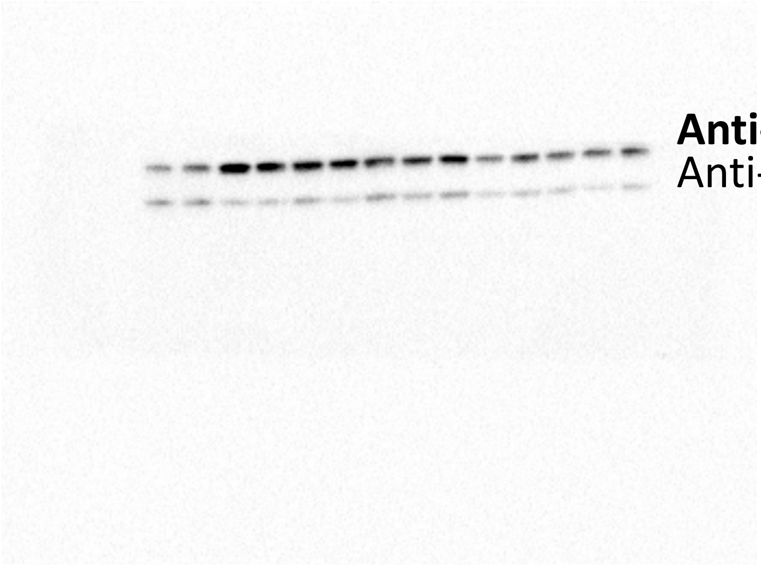

Anti-GAPDH

Anti-P-S6

C2 Vs P12

Figure S3C (replica 2)

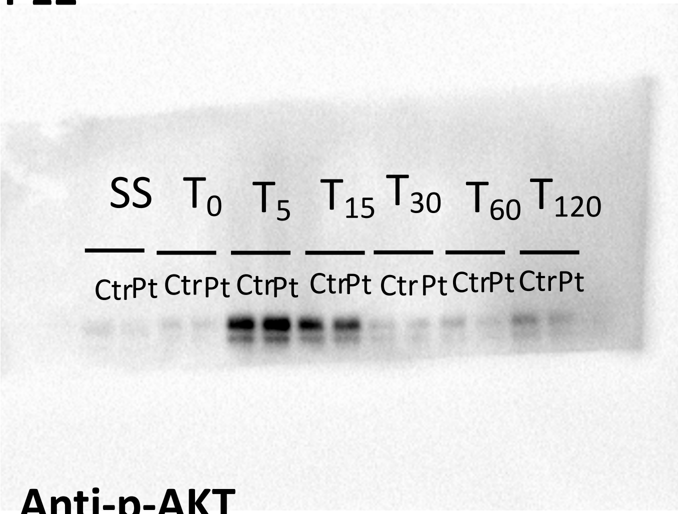

Anti-p-AKT

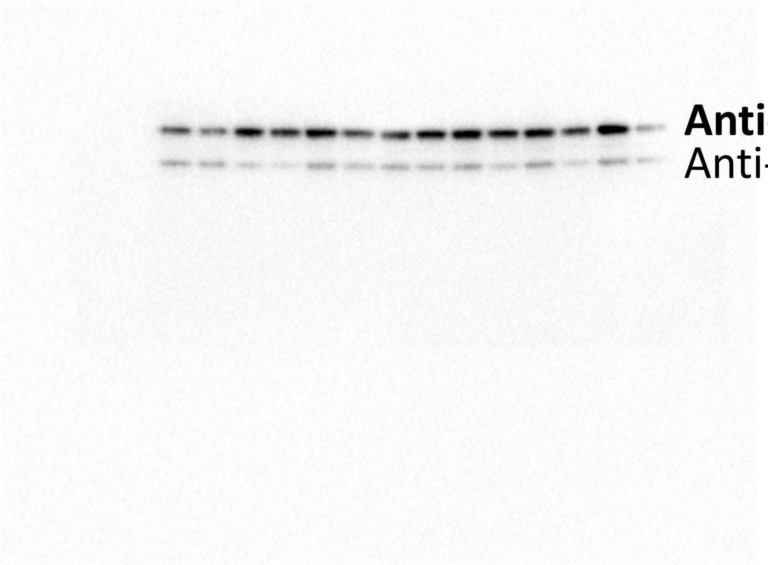

Anti-GAPDH

Anti-P-S6

N=1

Figure 3J

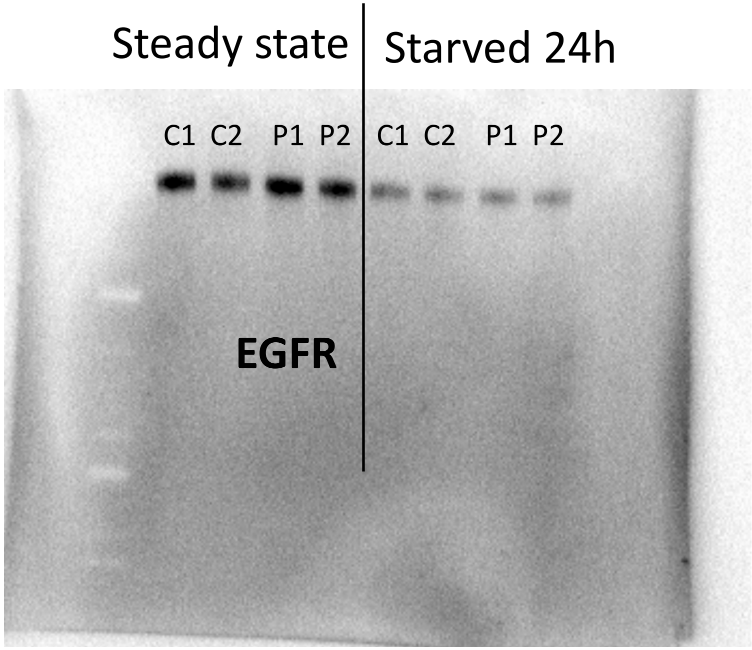

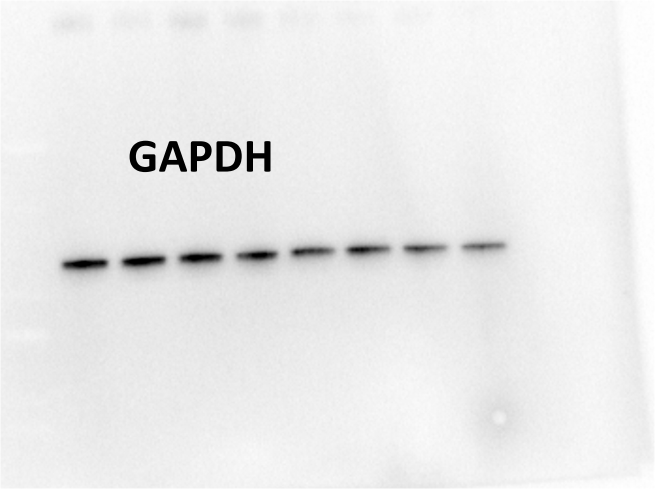

Figure 3J

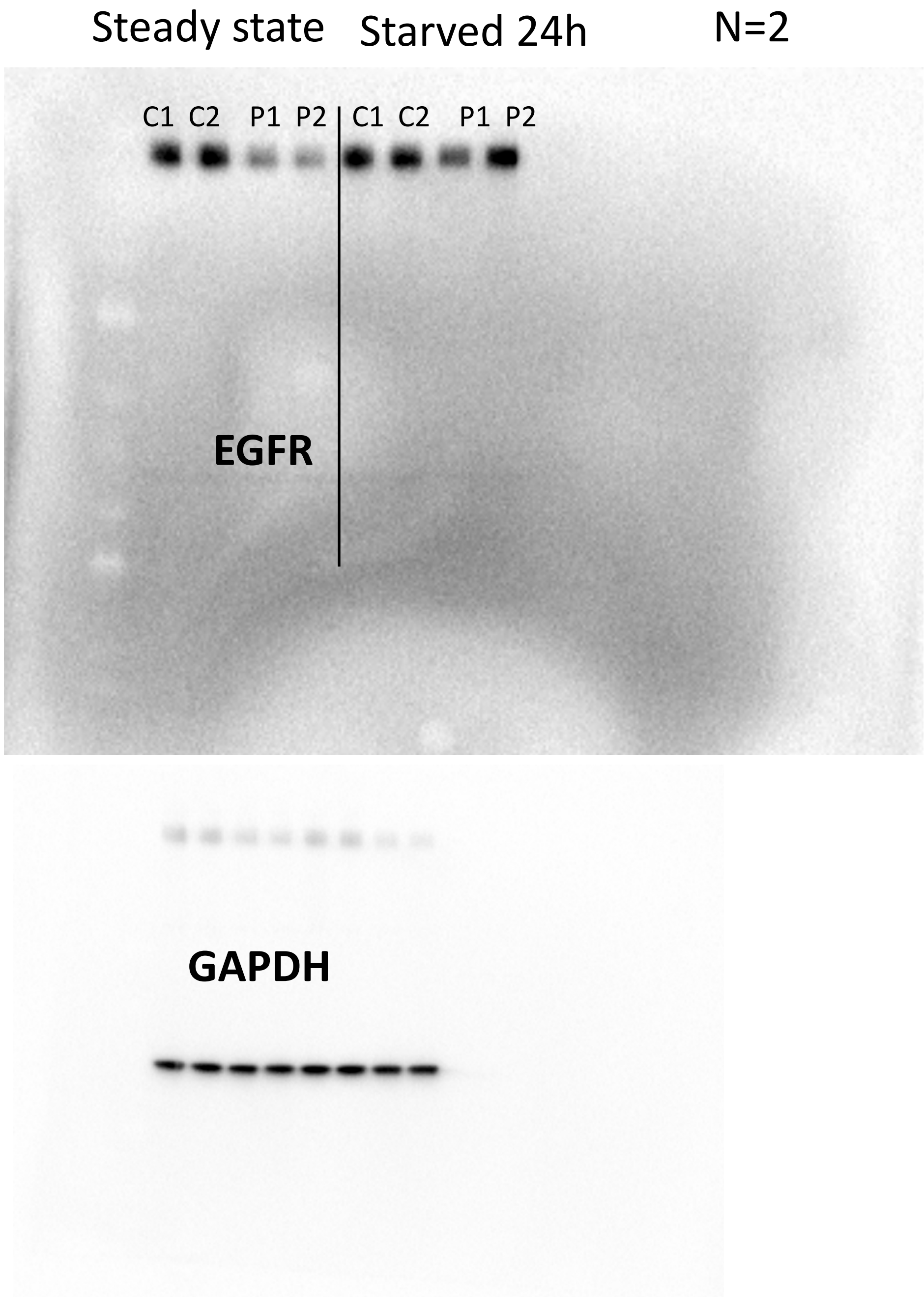

Figure 3J

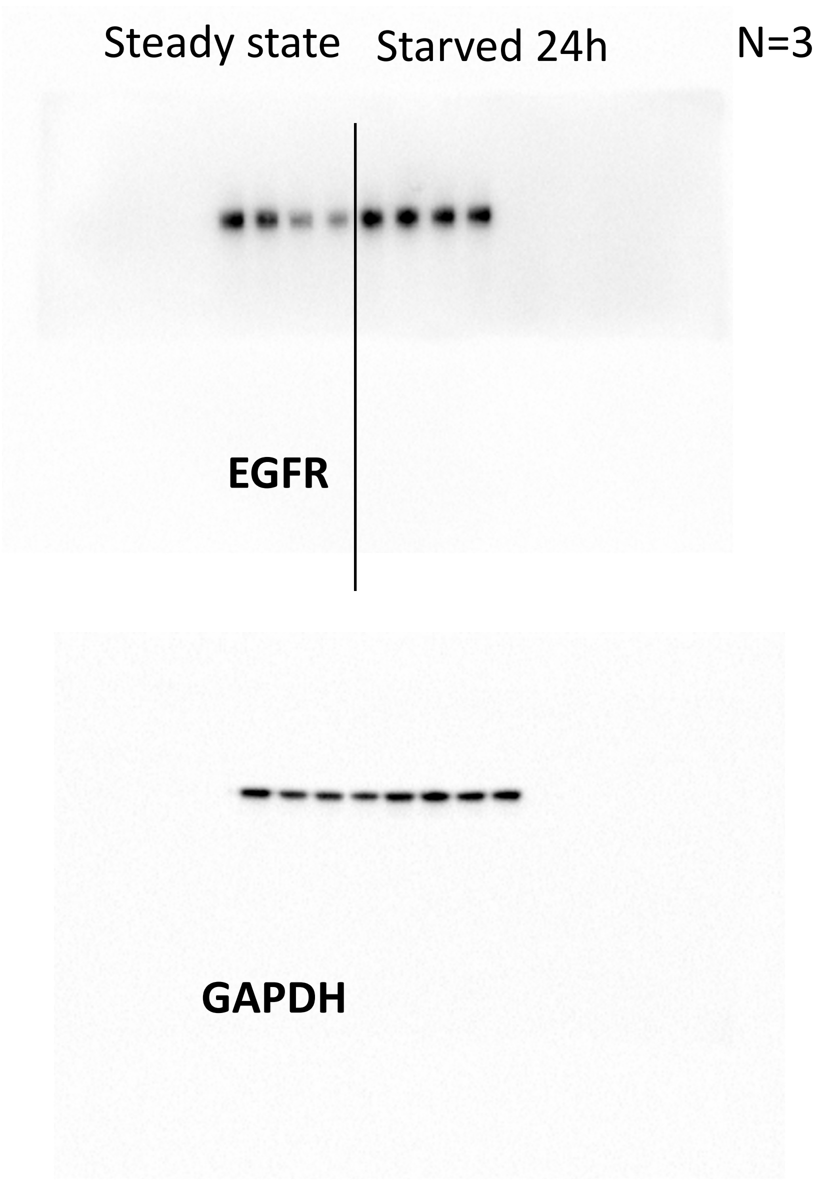

Figure 3J

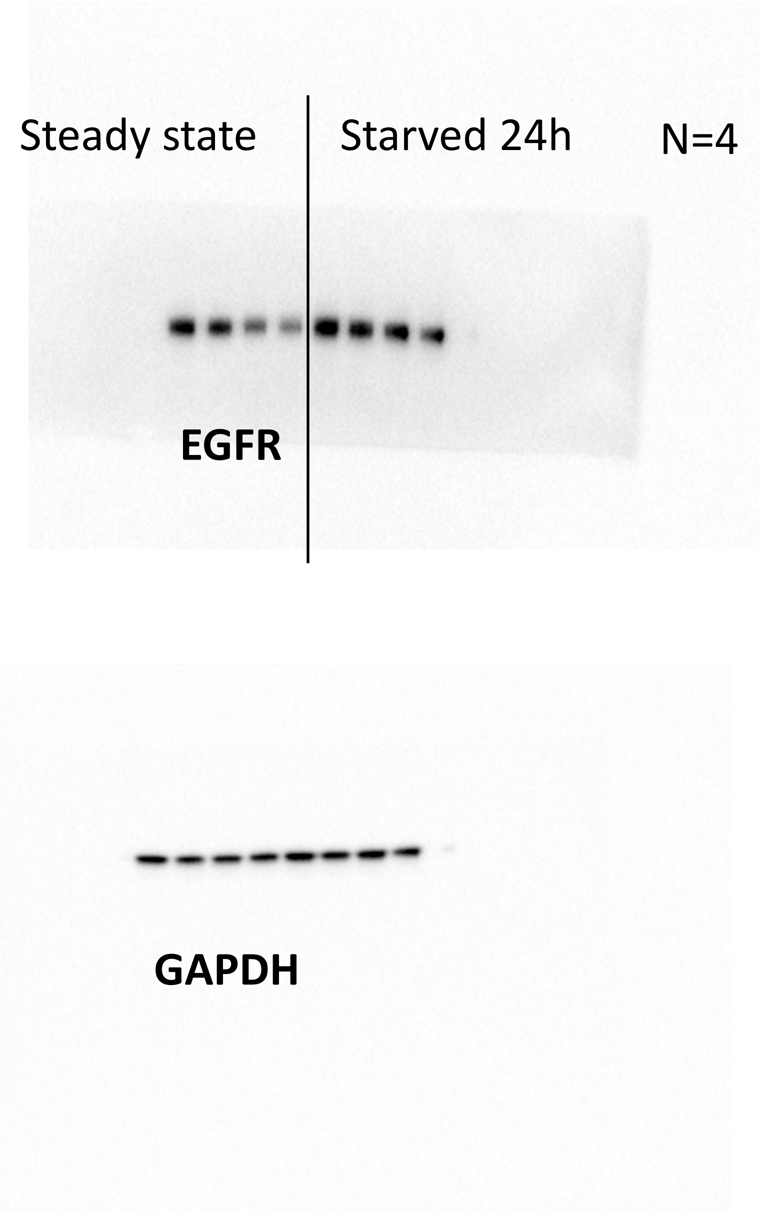

Supplement: Supplementary file 4 — Source Data [file 41467_2021_27705_MOESM4_ESM.zip › Supplementary Information_WB membranes.pdf]
